# Cystoscopic evaluation and clinical phenotyping in interstitial cystitis/bladder pain syndrome

**DOI:** 10.4274/jtgga.galenos.2018.2018.0102

**Published:** 2019-05-28

**Authors:** Ömer Acar, Tufan Tarcan

**Affiliations:** 1 Department of Urology, Koç University School of Medicine, İstanbul, Turkey; 2 Department of Urology, Marmara University School of Medicine, İstanbul, Turkey

**Keywords:** Bladder pain, cystoscopy, hydrodistension, biopsy, phenotyping

## Abstract

Herein, we aimed to review, report, and discuss the role of cystoscopy and clinical phenotyping in interstitial cystitis/bladder pain syndrome (IC/BPS). For this purpose; a comprehensive nonsystematic review of the relevant literature was conducted. We reviewed articles published in English and indexed in the PubMed, Embase, and Google Scholar databases. Original manuscripts, review articles, case series, and case reports were taken into consideration. Data regarding the indications for, technique, and possible findings of cystoscopy with hydrodistension (HD) and biopsy, as well as clinical implications of cystoscopic information and the concept and use of clinical phenotyping within the context of IC/ BPS were extracted and discussed. IC/BPS is diagnosed based on symptomatic assessment and exclusion of confusable diseases. There is no universal agreement upon the evaluation and diagnostic algorithm of IC/BPS. The majority of the guidelines recommend cystoscopy with HD and biopsy as a diagnostic prerequisite. Various different techniques have been described for cystoscopy with HD. General or epidural anesthesia is more commonly preferred and advocated while assessing endoscopic alterations in patients suspected of having IC/BPS. Cystoscopy with HD and biopsy enables more objective exclusion of confusable diseases. It also provides the basis of the European Society for the Study of Interstitial Cystitis classification. Patients with IC/BPS who demonstrate positive cystoscopic (glomerulations and/or Hunner lesion) and histologic findings have a more severe symptomatology and may benefit from lesion-targeted endoscopic treatments. Clinical phenotyping has been implemented for IC/BPS and may be used for individualized assessment and treatment.

## Introduction

Interstitial cystitis/bladder pain syndrome (IC/BPS) is a chronic disorder of unknown etiology and is one of the most debilitating conditions in urologic practice. It is characterized by pain, pressure or discomfort perceived to be related to the urinary bladder, associated with lower urinary tract symptoms of more than six weeks’ duration, in the absence of any identifiable cause such as infection (1,2). IC/BPS can be recognized by the presence of consistent symptoms and signs. Disorders that may lead to a similar symptomatology should be excluded in order to confirm the diagnosis of IC/BPS (2).

There are significant variations regarding the evaluation and diagnosis of IC/BPS (3). The role of cystoscopy in the diagnosis and classification of IC/BPS has long been a matter of debate, with some authorities such as the European Society for the Study of Interstitial Cystitis (ESSIC) indicating cystoscopy as a diagnostic prerequisite, whereas some others, such as the American Urological Association (AUA) reserve it for complex cases (4).

Clinical phenotyping, which categorizes the disorder according to the presence or absence of clinically relevant domains, has been implemented in IC/BPS after its success for chronic prostatitis/chronic pelvic pain syndrome (CP/CPPS) in an effort to optimize diagnosis and treatment (5). The main purpose of phenotype mapping in IC/BPS is to better understand the multifactorial etiology of the disorder and enable multimodal and phenotype-directed targeted therapy (6).

Herein, we review and discuss the contemporary English literature about cystoscopic evaluation and clinical phenotyping in IC/BPS.

### Indications for cystoscopy with hydrodistension and biopsy in IC/BPS

Indications for cystoscopy within the context of IC/BPS evaluation and management exhibit considerable variation. The National Institute of Diabetes and Digestive and Kidney Diseases (NIDDK) established cystoscopic discovery of glomerulations or Hunner lesions as an unchallenged diagnostic criterion for IC/BPS (7). However, NIDDK criteria were used mainly for the purpose of standardization in scientific studies and the strict application of these criteria would miss a significant proportion of patients who actually have IC/BPS (8). Many experts agreed that the absence of glomerulations or Hunner lesions did not rule out IC/BPS (9).

The ESSIC proposal highlighted the importance of excluding confusable diseases (such as carcinoma in situ) as the cause of symptoms and indicated cystoscopy under anesthesia with hydrodistension (HD) and eventual biopsy as a diagnostic prerequisite (2). Furthermore, cystoscopic and histopathologic findings would enable further documentation and classification of IC/BPS (2). The European Association of Urology (EAU) (10) and the Japanese Urological Association guidelines (11), conjoint expert opinions from East Asia (12), and the Bladder Pain Syndrome Committee of the International Consultation on Incontinence (13) follow the recommendations of the ESSIC. Conversely, the AUA guidelines do not indicate cystoscopy as an integral part of the initial diagnostic evaluation for IC/BPS (1).

### Technique of cystoscopy + hydrodistension in IC/BPS

Similar to its indications, the technical protocol of cystoscopy and HD in IC/BPS is subject to considerable variation and lacks consensus. The NIDDK recommended cystoscopy and HD to be performed under anesthesia, at a pressure of 80-100 cm H20, lasting 1-2 minutes, and up to 2 cycles. The presence of Hunner lesions or glomerulations that are diffuse in at least three quadrants with ten glomerulations per quadrant were considered positive findings in favor of IC/BPS (14). The ESSIC and EAU guidelines did not specify technical details about the cystoscopic evaluation for IC/BPS (2,10). According to the AUA guidelines, cystoscopy and HD should be performed under anesthesia, at a pressure of 60-80 cm H20, and be no longer than 10 minutes when the aim is therapeutic (1). The Japanese guidelines recommended lumbar anesthesia at the level of T6 during cystoscopy, with 80 cm H20 pressure, and to stop the infusion when the volume is between 800-1000 mL despite low pressures (11).

Apart from the guideline recommendations, some authors have proposed individual protocols. Turner and Stewart suggested a pressure of 100 cm H20 with a maximum infused volume of 1000 mL, and the distension being maintained for 1 minute. According to their technique, bladder cycling should not be repeated more than 5 times and cystoscopic assessment should be performed ideally at the initial and last distensions (15).

According to Nordling et al. (16), possible urethral urine leaks around the cystoscope should be blocked digitally. They also suggested that the bladder should be filled with a pressure of 80 cm H20 until the infusion stops dripping, without any specification about the volume limit. Emptying should be started after waiting for 3 minutes with the bladder fully distended. During filling and emptying, which can be repeated one more time, endoscopic assessment is performed. However, they recommend not to reach the maximum capacity during the second cycle to better visualize lesions and optimize tissue sampling (16).

The majority of the published series about IC/BPS stated general or spinal anesthesia as the preferred and recommended type of anesthesia to be applied during cystoscopy with HD. However, some investigators suggested that glomerulations or Hunner lesions could be visualized under local/regional anesthesia (17). Yamada et al. (18) supported the feasibility of epidural anesthesia in an effort to perform additional HDs on the next day following the initial cystoscopy +HD. Aihara et al. (19) used local anesthesia via intravesical administration of lidocaine 10 minutes prior to the start of the infusion, which was terminated when the patient reported intolerable pain or other local symptoms. They reported favorable results in terms of the safety and efficacy of this approach (19).

### Cystoscopic findings in IC/BPS

**Figure 1:**
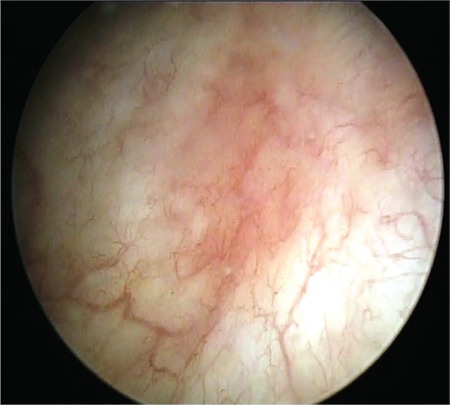
Cystoscopic view of Hunner lesion (courtesy of Dr. Tufan Tarcan)

Hunner lesions and glomerulations represent the most characteristic findings that might be encountered during the cystoscopic evaluation for IC/BPS. Hunner lesions were initially called ulcers. However, it is actually an inflammatory lesion that ruptures through the mucosa and submucosa when the bladder is distended. Hence, the suffix ‘lesion’ would more precisely define its characteristics. Hunner lesions encompass tiny vessels radiating towards a central scar, which is covered by coagulum. When they rupture upon bladder distension, petechial oozing of blood occurs in a waterfall manner (Figure 1) (2). Hunner lesions are not common, with only around 10-15% of patients with IC/BPS showing consistent cystoscopic signs (19,20,21). Narrow band imaging, which helps to distinguish the vascularity of a given bladder mucosal abnormality, has been proposed as an aid to better identify Hunner lesions endoscopically (22). However, more studies are needed to advocate its routine use for this purpose.

Glomerulations are a separate entity and they are defined as small submucosal petechial lesions that become visible after bladder HD (23). They are classified into five grades according to the extent of submucosal bleeding and the presence/absence of mucosal disruption (16). The term ‘glomerulation’ was introduced by Walsh who linked these mucosal changes to early stage disease and also highlighted that they were not pathognomonic for IC because other bladder pathologies, such as dyskinesia, might lead to similar alterations in the bladder mucosa (24). Being mainly related to IC/BPS, glomerulations are neither specific nor sensitive enough when used solely for diagnostic purposes. Patients with chronic inflammation of the urothelium, urinary tract stone disease, and benign prostate hyperplasia can exhibit endoscopic signs consistent with glomerulations (25,26). Furthermore, Waxman et al. (27) showed that glomerulations could even be discovered in otherwise healthy women. On the contrary, the proportion of patients with a clinical diagnosis of IC/BPS but with no cystoscopic changes can be in the range of 24-34% (28,29).

### Classification of ic/bps according to findings at cystoscopy with hydrodistension and biopsies

According to the ESSIC, cystoscopy and HD with biopsy is an integral part of the diagnostic evaluation for IC/BPS. Cystoscopic positive signs in favor of IC/BPS are glomerulations grade 2-3 or Hunner lesions or both. Infiltration of inflammatory cells and/or formation of granulation tissue and/or overexpression of mast cells and/or intrafascicular fibrotic changes represent the histopathologic findings that are interpreted in favor of IC/BPS (2). IC/BPS subtypes are defined on the basis of cystoscopic and histopathologic findings (Table 1). If cystoscopy or biopsy are not performed, then the letter X is assigned. Biopsy findings are categorized as follows: normal (A), inconclusive (B), and positive (C). Cystoscopic findings are interpreted as follows: normal (1), glomerulations (2), and Hunner lesion (3). This type of classification could not be possible if only clinical findings were used. Moreover, such a distinction would have implications regarding prognosis and treatment outcome.

### Clinical implications and correlations regarding cystoscopy with hydrodistension and biopsy findings in IC/BPS

The clinical relevance of IC/BPS subtypes has long been questioned. However, the information gathered through cystoscopic examinations and histopathological assessments of bladder biopsy samples in IC/BPS offer several advantages regarding optimizing patient management and treatment outcomes. First of all, IC/BPS is essentially a diagnosis of exclusion. Cystoscopy with HD +/- biopsy offers the unique opportunity to exclude some confusable diseases such as carcinoma in-situ and bladder stones in a more reliable manner (2,30). 

Moreover, patients with Hunner lesion IC/BPS may benefit from targeted endoscopic interventions. Transurethral resection of Hunner lesions has been associated with symptomatic improvement rates in the range of 90% (31,32). Hunner lesion-directed endoscopic treatment options were further enriched by studies investigating the potential utility of Nd: YAG laser, electrocoagulation, and instillation of triamcinolone (33,34,35), all of which reported impressive improvement rates ranging from 70-90%. This therapeutic benefit would not have been possible if these patients were not identified via cystoscopy +/- biopsy. It has been shown that a reliable distinction between Hunner lesion IC/BPS and non-Hunner lesion IC/BPS is not possible via clinical assessment only (36,37). Furthermore, cystoscopy under local anesthesia can be used to monitor the effect of bladder distension and emptying on pelvic symptoms. Despite the limitation that might be induced by pain and/or discomfort, functional bladder capacity can also be assessed in the same setting (17).

Regarding the correlation between cystoscopy and clinical findings; recent studies have shown that patients with Hunner lesion IC/BPS are more severely symptomatic than patients without Hunner lesions. In their study in which 393 patients with IC/BPS (55% with type 3C) were enrolled, Logadottir et al. (38) investigated the potential clinical similarities and dissimilarities between the main disease subtypes. They found that patients with type 3C disease were older (62 vs 42 years, p<0.001), with a lower average maximal voided volume (206 vs 289 mL, p<0.001), and a lower average bladder capacity under anesthesia (459 vs 743 mL, p<0.001). Boudry et al. (39) assessed the use of a bladder diary for discriminating between Hunner lesion vs nonHunner lesion IC/BPS. For this purpose, they used the clinical data of 54 consecutive (39 women and 15 men) patients and discovered an association between the bladder diary parameters and cystoscopic alterations such that those with positive cystoscopic findings had lower functional bladder capacities, an increased rate of frequency and nocturia, and greater relief of symptoms upon voiding when compared with those who had normal cystoscopic findings (39). Ahn et al. (40) studied the differences between Hunner lesion IC/BPS and nonHunner lesion IC/BPS with regard to bladder diary findings and urodynamic parameters in a cohort of 55 female patients. According to bladder diary data, the frequency of micturitions was higher in the Hunner lesion group (16.65 vs 12.53, p=0.045) together with a smaller amount of maximal voided volume (143.48 vs 244.53 mL, p<0.001). Regarding the urodynamic recordings, the desire to void and maximum cystometric bladder capacity (MBC) (182.09 vs 286.59 mL, p<0.001) corresponded to significantly lower volumes in the Hunner lesion group. The authors identified cut-off values for urodynamic parameters to predict the presence of Hunner lesions on cystoscopy and suggested that endoscopic evaluation of the bladder should be offered to patients with a strong desire to void volumes ≤210 mL or with an MBC ≤236 mL (40).

Finally, cystoscopy is not a morbid procedure, having a fairly low incidence of complications. Relatively few publications have focused on the complications of cystoscopy and HD performed primarily within the context of IC/BPS management. Apart from anecdotal reports of bladder rupture, bladder necrosis, and acute pyelonephritis, the procedure seems to be safe and well tolerated (41,42).

### Clinical phenotyping in IC/BPS

IC/BPS is a disorder without a universal agreement upon its etiology, diagnostic algorithm, and management strategy. IC/BPS may be regarded as a component of a more generalized somatic problem, reflections of which may affect the urinary bladder and other pelvic organs via several proposed mechanisms. The release of mediators such as leukotriene from activated mast cells located close to the neural/perineural structures along the bladder wall is the most widely studied etiopathogenetic explanation for IC/BPS (43). 

Diverse clinical phenotypes might be encountered within the context of IC/BPS (44,45). The concurrent existence of IC/BPS with other chronic pain and symptom-based syndromes have been documented (45,46). 

The main aim of phenotype mapping for IC/BPS has been to provide more individualized and phenotype-directed clinical assessment and treatment. The urinary symptoms, psychosocial dysfunction, organ specific findings, infection, neurologic/systemic and tenderness of muscle (UPOINT) schema, which provides better classification and treatment for CP/CPPS (45), has been extrapolated for IC/BPS. Nickel et al. (44) categorized patients with IC/BPS into 6 domains: the urinary domain, which includes patients with bothersome lower urinary tract symptoms; the psychosocial domain, which is characterized by patients with clinical depression or an identifiable maladaptive coping mechanism; the organ-specific domain, which mainly comprises patients with typical cyclic pain provoked by bladder filling and temporary relief with voiding and/or demonstrating positive cystoscopy + biopsy findings; the infection domain, which consists of patients with urine culture-documented urinary tract infections within the last 2 years that provoked/exacerbated baseline symptoms, the neurologic/systemic domain hallmark of which being prior diagnoses of disorders involving some degree of neuropathy or neural upregulation (e.g. irritable bowel syndrome, fibromyalgia, chronic fatigue syndrome, vulvodynia); and the tenderness domain, which includes patients who demonstrate trigger point tenderness during physical examination. Patients with more UPOINT-positive domains experienced more severe symptoms of longer duration as determined by the interstitial cystitis symptom index (ICSI) (44). Treatment options that can be recommended based upon the UPOINT classification are summarized in Table 2 (47).

Doiron et al. (17) investigated the use of clinical phenotyping in distinguishing Hunner lesion IC/BPS from nonHunner lesion IC/BPS in their cohort composing 359 patients (12.3% with Hunner lesions) with documented cystoscopic findings. The Hunner lesion group reported higher ICSI scores together with higher rates of pain, frequency, and nocturia when compared with the nonHunner group. However, the difference between the two groups was not statistically significant in terms of the number and distribution of UPOINT phenotypes. Despite the lack of statistical significance, there was a trend towards a more prevalent urinary domain in the Hunner lesion IC/BPS group (17). The authors concluded that patients with Hunner lesion IC/BPS could not be identified by clinical phenotyping alone and cystoscopy was inevitable for such a discrimination.

IC/BPS is diagnosed based on symptomatic assessment and exclusion of confusable diseases. There is a lack of consensus regarding the evaluation and diagnostic algorithm of IC/BPS. European and Asian guidelines recommend cystoscopy with HD and biopsy as a diagnostic prerequisite. On the other hand, cystoscopic examination is not a routine part of the diagnostic evaluation according to the AUA. Considerable variation exists about the technique of cystoscopy with HD. General or epidural anesthesia is usually preferred while examining the bladder in patients with clinical signs of IC/BPS. However, certain authorities support the feasibility and have highlighted the advantages of local anesthesia for the same purpose. Cystoscopy with HD and biopsy enables exclusion of the confusable diseases in a more reliable manner. It also forms the basis of the ESSIC-proposed classification of IC/BPS. The identification of patients who demonstrate positive cystoscopic signs and histopathologic alterations in favor of IC/BPS might have implications regarding treatment outcome because lesion-targeted endoscopic treatment has yielded promising results. Patients with Hunner lesion IC/BPS tend to be older with a more severe symptomatology in terms of pain and lower urinary tract symptoms when compared with those with nonHunner lesion IC/BPS. Clinical phenotyping has been implemented in IC/BPS. Categorizing patients according to UPOINT domains might enable individualized treatment.

## Figures and Tables

**Table 1:**
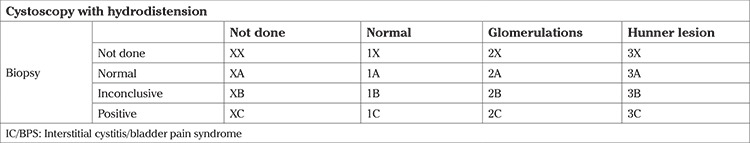
Classification of types of IC/BPS according to findings at cystoscopy with hydrodistension and biopsies

**Table 2:**
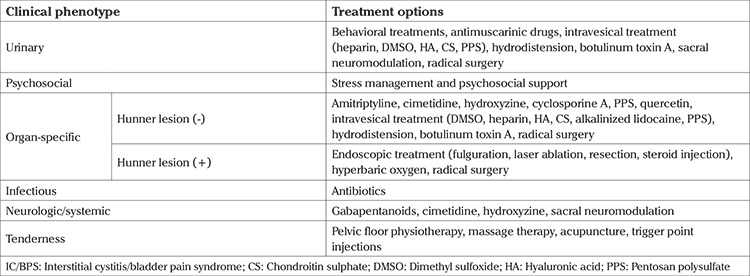
Treatment options which can be recommended based upon the predominant clinical phenotype of the patients with IC/BPS
